# Symptomatic perineural cyst after spontaneous subarachnoid hemorrhage: A case report

**DOI:** 10.1097/MD.0000000000025587

**Published:** 2021-04-23

**Authors:** Jongpil Eun, Youngmin Oh

**Affiliations:** Department of Neurosurgery, Biomedical Research Institute, Jeonbuk National University Medical School and Hospital, Jeonju, South Korea.

**Keywords:** perineurial cysts, sciatica, spinal cyst, subarachnoid hemorrhage

## Abstract

**Rationale:**

Tarlov or perineurial cysts are nerve root lesions often found in the sacral region. Most perineural cysts (PCs) remain asymptomatic throughout a patient's life. While their pathogenesis is still unclear, trauma resulting in hemorrhaging into subarachnoid space has been put forward as a possible cause of these cysts. Recently, we worked with a patient experiencing symptomatic PCs after spontaneous subarachnoid hemorrhage.

**Patient concerns:**

A 45-year-old man had a coil embolization procedure performed after being diagnosed with a subarachnoid hemorrhage from a ruptured anterior communicating artery. His symptoms were relieved after the procedure, but 7 days later he reported worsening pain in the left perineal area. The pain was intermittent at its onset and exacerbated by sitting, walking, and coughing.

**Diagnoses:**

Two weeks after the embolization procedure, a lumbar spine MRI revealed 2 PCs at the S1 and S2 level affecting the left S2 root with high signal intensity in T2 and T1 images, suggestive of bleeding within the cyst.

**Interventions:**

We operated using a posterior approach. Cyst fenestration was done after S1 laminectomy. We aspirated approximately 1 cc of old blood.

**Outcomes:**

His pain was relieved immediately after cyst removal and no neurologic deterioration occurred during the postoperative period.

**Lessons:**

Subarachnoid hemorrhage can be the source of the development of pain from asymptomatic PCs, making them symptomatic. Surgical extirpation is 1 treatment option for these symptomatic PCs.

## 1 Introduction

Tarlov or perineural cysts (PCs) are benign, extradural spinal cysts containing cerebrospinal fluid (CSF).^[1,2]^ The cysts arise at the junction of the dorsal root ganglion and the posterior nerve root. They are most often found in the sacral region, developing between the endoneurium and perineurium.^[3,4]^ The presence of a communication between the cyst and thecal sac differentiates PCs from other cystic lesions. The prevalence of PCs have been estimated at 1.5% to 4.6% of the population. The majority of these lesions are asymptomatic, but less than 1% of these cysts may cause clinical symptoms. This depends on the location of the cyst in the spinal canal and the type of nerve roots it is compressing.^[5]^ PCs can be symptomatic if their internal pressure is substantial, and they can exert pressure on neural tissue as well as surrounding bone.^[5]^ The most common presenting symptoms include lower-back pain, sacrococcygeal pain, perineal pain, sciatica, motor deficits, cauda-equina syndrome and intrathecal hypotension. The symptoms are often intermittent at onset and are most frequently exacerbated by standing, walking, coughing, postural changes and Valsalva maneuvers such as sneezing or straining to defecate, all of which elevate the CSF pressure. Perineural cysts are located by using neuroimaging, including lumbosacral or pelvic magnetic resonance imaging (MRI) and computed tomography (CT) myelography.

Previous authors have suggested that the enlargement of PCs through a ball-valve mechanism and the resultant compression of the adjacent neural or bony structures is one of the sources of the pain, but the reason why some asymptomatic PCs progress to cause symptoms is still unclear.^[6–8]^ Hemorrhaging has been considered as one possible cause of PC-related symptoms. The origin of the cysts has been thought to be trauma with resultant hemorrhage into subarachnoid space. Symptomatic PCs following spontaneous subarachnoid hemorrhage (SAH), however, are extremely rare. Recently, we work with a patient experiencing symptomatic PCs following spontaneous SAH and performed surgical excision removing the PCs and successfully relieving painful symptoms. Patient has provided informed consent for publication of the case.

## 2 Case report

**Figure 1:**
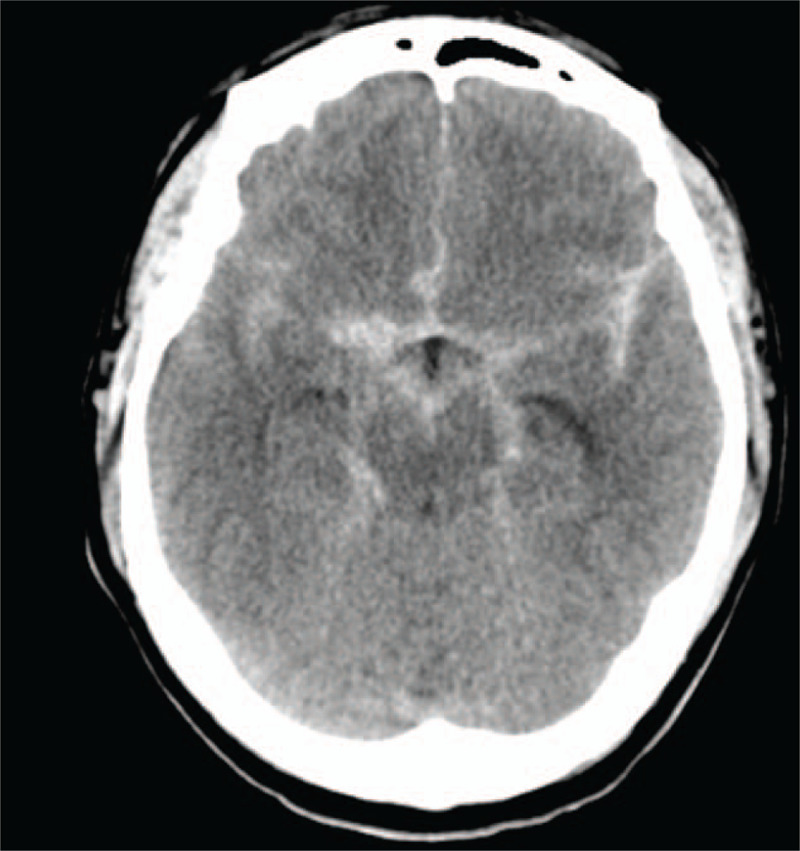
Brain computed tomography taken at the emergency room revealed SAH in the bilateral Sylvian fissure, basal cistern and interhemispheric fissure. SAH = subarachnoid hemorrhage.

**Figure 2:**
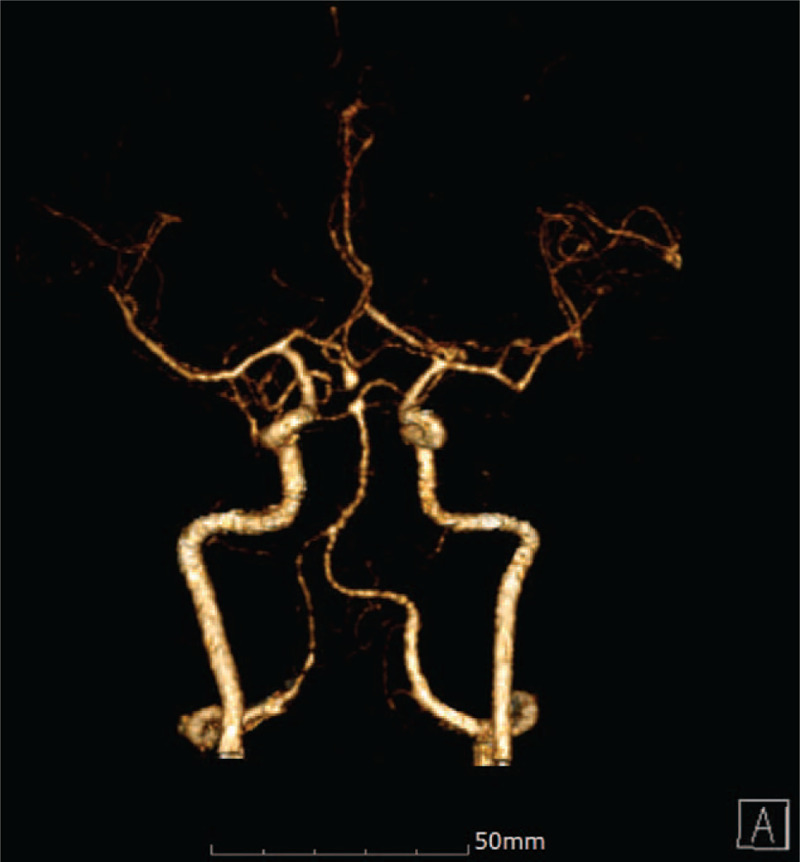
Three-dimensional brain CT angiography showed a right feeding anterior communicating artery aneurysm. CT = computed tomography.

**Figure 3:**
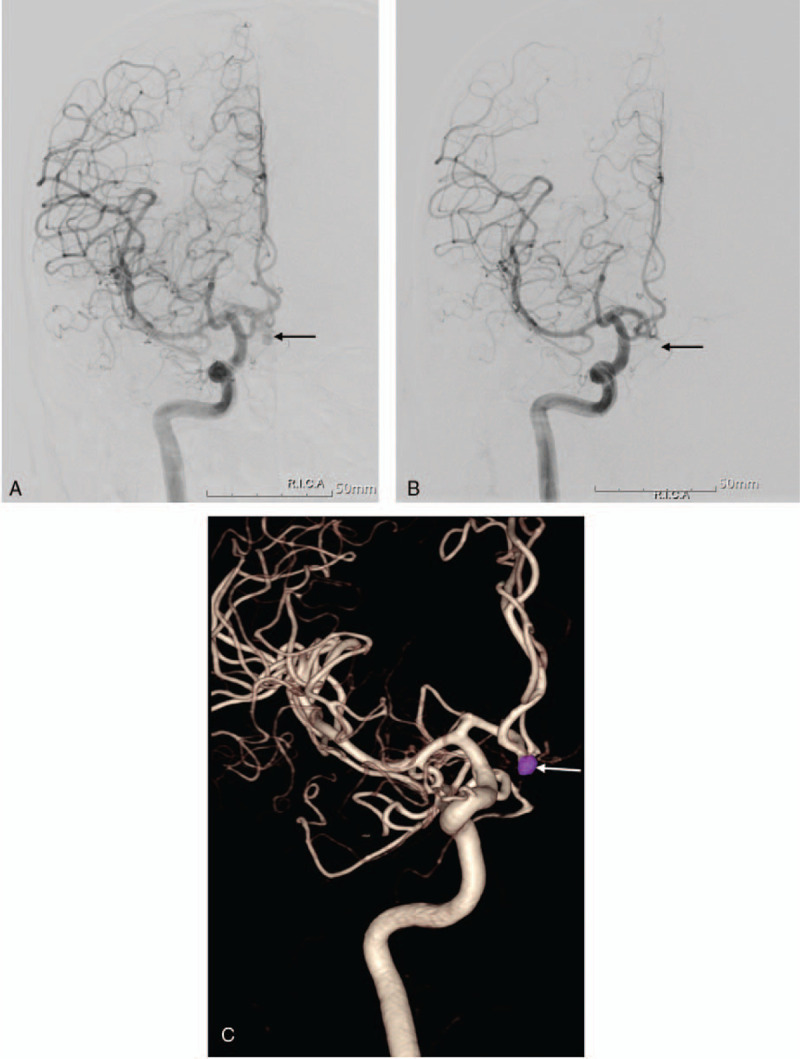
(A) Emergent cerebral angiography showed an aneurysm of the anterior communication artery (arrow). (B) Cerebral angiography after endovascular coil embolization revealed complete occlusion of the aneurysm (arrow). (C) Reconstruction image of post-embolization showed a completely packed coil in the aneurysm (arrow).

**Figure 4:**
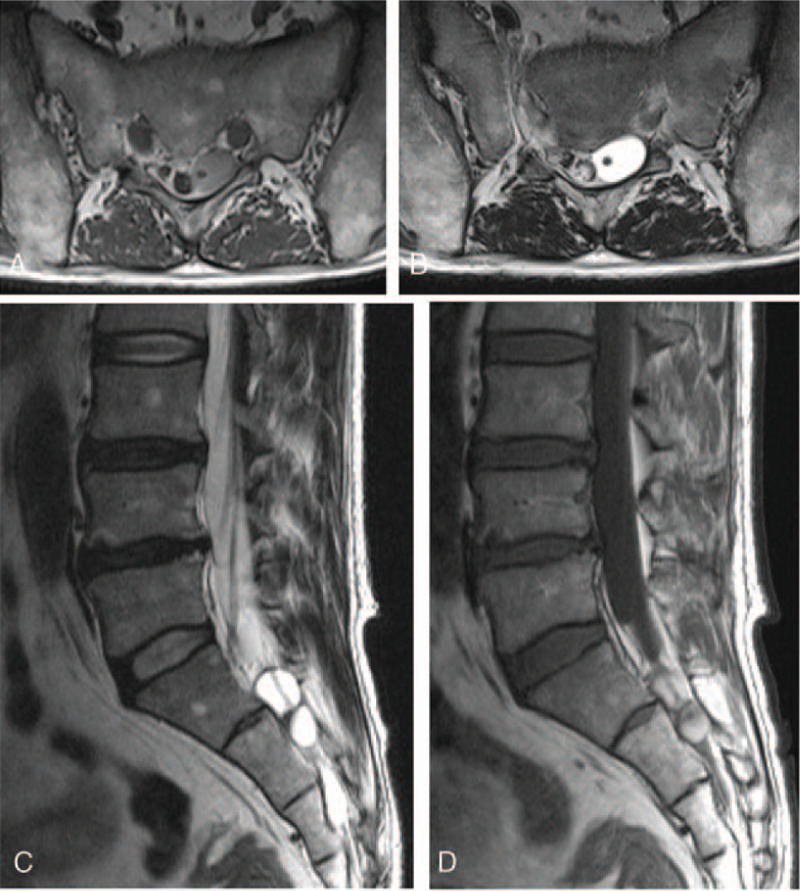
Non-enhanced T1-weighted (A) and T2-weighted (B) axial MRI showed the perineural cyst located to the left side and encompassing the nerve root. The left nerve root was smaller than the right S2 root because it was compressed by increased intracystic pressure. (C) T2-weighted sagittal MRI revealed 2 separated perineural cysts with high signal intensity at S1 and S2 levels. There was a nerve root inside the cyst (arrow). (D) Non-enhanced T1-weighted sagittal MRI showed mixed high and low signal intensity with a fluid-fluid level within the cyst, suggestive of intracystic hemorrhage. MRI = magnetic resonance imaging.

A 45-year-old man was transferred to the emergency room, reporting a severe headache as well as sudden onset nausea. Upon neurological examination, he was found to be drowsy, but was otherwise normal. Brain CT revealed SAH in the bilateral Sylvian fissure, basal cistern and interhemispheric fissure (Fig. 1). Three-dimensional brain CT angiography showed a right feeding anterior communicating artery aneurysm (Fig. 2). Emergent cerebral angiography and endovascular coil embolization were performed (Fig. 3). After embolization, the headache was alleviated and the patient's status improved each day. Soon after recovery, he reported worsening pain in the left perineal area. The pain was intermittent at onset and aggravated by sitting down, walking, straining and coughing. Although bed rest relieved the pain, he had difficulty falling asleep due to the pain. He had no history of previous back problems or recent trauma. Upon a second neurological examination, no abnormalities were found. Two weeks after embolization, lumbar spine MRI revealed 2 separated PCs at the S1 and S2 level affecting the left S2 root with high signal intensity in T2 and T1 images, suggestive of a hemorrhage within the cysts (Fig. 4). The diameter of the larger cyst was 1.4 cm. The left S2 root was smaller than the right S2 root because it was compressed by increased intracystic pressure. Given that his symptoms were not relieved with analgesics or a caudal block, and the radiological evidence of PCs, we decided to perform cyst fenestration and imbrication.

**Figure 5:**
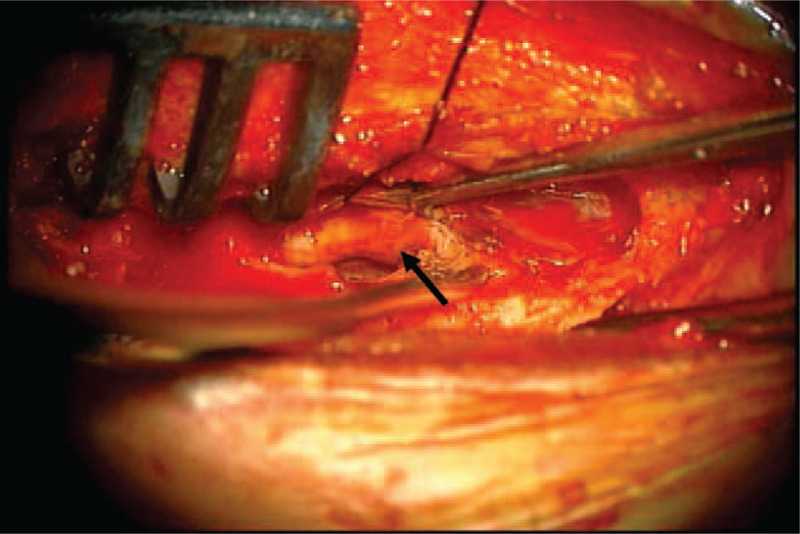
On the operative field, we could find the S2 nerve root inside the cyst after hematoma removal (arrow).

Under general anesthesia, we performed partial laminectomies at S1 and S2 via a posterior approach. The 2 separate cysts were tense and swollen. When we incised the cyst, dark old blood drained out and the cyst contracted. After the hematoma evacuated, we could find the S2 nerve root inside the cyst (Fig. 5). The patient's pain was relieved immediately after cyst fenestration and imbrication. In a follow-up 5 years after the surgery, the patient remained symptom free with no complications.

## 3 Discussion

Most PCs remain asymptomatic throughout the patient's life.^[6]^ The reason why these asymptomatic PCs become symptomatic is uncertain. Although the etiology is still unclear, micro-communication with the subarachnoid space at the dural sleeve of the nerve root may function as a valve, allowing CSF influx and restricting CSF efflux, causing formation of the cysts.^[2,9]^ Some researchers have postulated that cyst formation may result from a number of factors including: spontaneous or traumatic hemorrhage into the subarachnoid space, ischemic degeneration, proliferation of arachnoid membranes or inflammation of the nerve root with subsequent edema.^[1,3,10]^ Still others have suggested that PCs could be developmental or congenital in origin.^[2,11]^ While the causes and origins of the cysts are still debated, we do know how they grow and begin to cause symptoms. With stenosis of the ostium of the nerve root sheath, the cyst neck serves as a valve. CSF can flow into the cyst through the ostium by hydrostatic pressure, arterial pulsation and a patient's postural changes, but outflow is restricted because of a ball-valve effect.^[12]^ This ball-like mechanism causes the cyst to fill and expand in size, and may compress neighboring nerve fibers, resulting in neurological symptoms.^[13]^

With our patient, we speculated that intracystic hemorrhage occurred as a result of the SAH and then it induced an increase in intracystic pressure. After an aneurysmal rupture, intracranial SAH can migrate to the spinal subarachnoid space because the anterior and posterior spinal cisterns communicate through the foramen magnum with posterior fossa cisterns.^[14]^ Spinal SAH can then flow into the pre-existing PC due to arterial pulsation and pressure differences between spinal subarachnoid space and the PC. The hemorrhages result in increased hydrostatic CSF pressure, which leads to the infiltration of CSF and hemorrhaging into the pre-existing PC. As a result, intracystic pressure increases and nerve roots inside the cyst as well as near the cyst become compressed and symptomatic. The old blood that appeared from the PC of our patient could be evidence of the ball-valve mechanism in the cyst expansion. Transient stretching of the nerve root by hemorrhaging or irritation of the nerve root by an inflammatory reaction to blood products may be the source of the pain.

Numerous techniques for the management of symptomatic PCs have been described in the literature, with variable results. As Tarlov reported in his original article, he removed the domes of the cysts or completely excised the lesions along with the dorsal root ganglion. Since the publication of this article, several authors have proposed microsurgical cyst excision as well as cyst fenestration and imbrication as possible treatment options.^[15]^ There is no consensus, however, on the most effective treatment of symptomatic PCs. Cho et al. reported that conservative treatment should be given priority in symptomatic PCs with intracystic hemorrhage following SAH, because the intracystic hemorrhage is absorbed and the symptoms improve.^[14]^ Given the resistance of symptoms to medication and/or prior intervention, however, patients need to be offered the surgical option. For instance, Xu et al. reported poor outcomes in 2 patients treated with medication and physical therapy for symptomatic sacral PCs. Both patients symptoms worsened with time, and MRI revealed that the culpable cysts had increased in size within 5 years of initial diagnosis.^[16]^ Moreover, while temporary lumbar drainage can reduce symptoms by shrinking the sacral cyst, these symptoms can occur again after removal of drainage. For these reasons, symptomatic PCs should be surgically treated.^[17]^ Based on the notion that eliminating the intracystic pressure will resolve the pain, some authors reported that CT-guided aspiration of the cyst, which eliminates the need for general anesthesia and a long procedure, could be one of the treatment options for symptomatic PCs. The effectiveness of percutaneous drainage, however, is debatable. Voyadzis et al. did not recommend percutaneous aspiration of PCs because of the high rate of symptom recurrence and the low rate of improvement.^[3]^ Paulsen et al. reported that patients who underwent sacral meningeal cyst aspiration tend to accumulate CSF and become symptomatic again in 3 weeks to 6 months.^[5]^ In turn, they recommended aspiration should be used prior to considering open surgical decompression. Neulen et al and Guo et al have suggested that surgical treatment should be considered for cysts larger than 1 to 1.5 cm in size presenting with radicular symptoms, with the treatment being strongly correlated with excellent clinical outcomes.^[9,18]^ Recently, Sharma et al performed a meta-analysis of all the available reported cases of symptomatic PCs and concluded that surgical procedures are superior to percutaneous interventions in terms of resolution and long-term patient-reported outcomes.^[19]^ They further indicated that surgical procedures can be chosen for young healthy patients with better long-term cyst resolution but with an increased risk of postprocedural complications. Percutaneous techniques can be considered for elderly patients with multiple medical morbidities who are not fit to undergo surgical procedures involving general anesthesia and cannot withstand the postprocedural complications.

For our patient, we performed cyst fenestration and imbrication to address symptomatic PCs following SAH. The size of cyst was 1.4 cm and the patient had radicular symptoms that were not alleviated with conservative pain control treatment. During the operation, we performed a Valsalva maneuver that ensured there was no CSF leakage after cyst fenestration. In turn, we eliminated the possibility of the recurrence of the cyst or CSF leakage. Since the initial symptom of increased CSF hydrostatic pressure was eliminated after embolization, we were able to conclude that in the 5 years following the surgery there were no complications related to cyst excision or symptom recurrence. Fortunately, a positive clinical outcome was achieved without any complications.

## 4 Conclusion

In this report we discussed an extremely rear case of symptomatic PCs following SAH. Our case shows that SAH could be the triggering factor in causing an asymptomatic perineural cyst to become symptomatic. If symptoms are not relieved despite extensive conservative treatment, surgical cyst excision is unavoidable. Post-surgery our patient showed positive clinical results without any postoperative complications.

## Author contributions

**Conceptualization:** Youngmin OH.

**Data curation:** Youngmin OH.

**Funding acquisition:** Youngmin OH.

**Investigation:** Jongpil Eun, Youngmin OH.

**Methodology:** Youngmin OH.

**Writing – original draft:** Jongpil Eun, Youngmin OH.

**Writing – review & editing:** Jongpil Eun, Youngmin OH.
